# Internalization and Recycling of the HER2 Receptor on Human Breast Adenocarcinoma Cells Treated with Targeted Phototoxic Protein DARPinminiSOG

**Published:** 2015

**Authors:** O. N. Shilova, G. M. Proshkina, E. N. Lebedenko, S. M. Deyev

**Affiliations:** Shemyakin/Ovchinnikov Institute of Bioorganic Chemistry, Russian Academy of Sciences, Miklukho-Maklaya Str., 16/10, Moscow, 117997, Russia

**Keywords:** internalization, recycling, scaffold proteins, DARPin, targeted phototoxic protein, HER2

## Abstract

Design and evaluation of new high-affinity protein compounds that can selectively and efficiently destroy human cancer cells are a priority research area in biomedicine. In this study we report on the ability of the recombinant phototoxic protein DARPin-miniSOG to interact with breast adenacarcinoma human cells overexpressing the extracellular domain of human epidermal growth factor receptor 2 (HER2). It was found that the targeted phototoxin DARPin-miniSOG specifically binds to the HER2 with following internalization and slow recycling back to the cell membrane. An insight into the role of DARPin-miniSOG in HER2 internalization could contribute to the treatment of HER2-positive cancer using this phototoxic protein.

## INTRODUCTION

Monoclonal antibodies and their derivatives are widely used in clinical application for selective destruction of human tumors [1, 2]. At the same time, the development of new approaches (such as fully synthetic libraries, phage and ribosomal display) for *in vitro *generation of non-natural proteins with high affinity for a given target has led to the creation of non-immunoglobulin scaffold proteins with one whole framework, carrying altered amino acids that confer on protein variants specificity to different targets [3-5]. Scaffold proteins, having the same affinity and specificity, surpass their corresponding monoclonal antibodies in physical and chemical properties. They possess such desired properties as a small size, which enables efficient tissue penetration, rapid folding, high chemical, proteolytic and thermal stability and they do not tend to aggregate. A scaffold protein can be engineered to have a unique cysteine that facilitates further chemical conjugation to cytotoxic molecules, fluorophores and nanoparticles. Moreover, the absence of disulfides allows to expresse proteins in the cytoplasm of *Escherichia coli *and produce proteins with high affinity to selective target without animal immunization. These features give scaffold proteins advantages over antibodies being used as binding moieties in multifunctional compounds for the diagnosis and treatment of human diseases.

Alternative binding molecules include adnectins, affibodies, anticalins and proteins based on naturally occurring repeat proteins (ankyrin and tetratricopeptide repeats) [3-5].

In the laboratory of Dr. Pluckthun set-designed ankyrin repeat proteins (DARPins ) with high affinity for human epidermal growth factor receptor 2 (HER2 or ERBB2) have been developed [6].

The transmembrane receptor HER2 is overexpressed in 20–30% of breast and ovary tumors [7, 8]. High level of HER2 expression usually correlates with aggressive tumor phenotype and enhanced metastasis [9]. Because HER2 is expressed at relatively low levels in normal epithelial cells, it makes this receptor an attractive target in cancer therapy. In summary, the design and evaluation of novel, highly specific molecules capable of selectively killing cancer cells overexpressing HER2 remain an important task.

In this work, we used DARPin_9-29 as a targeting module to deliver the phototoxic protein miniSOG [10] to human breast adenocarcinoma HER2-positive cells.

The objective of this study was to assess the binding of the fusion protein DARPin-miniSOG to a HER2 overexpressing tumor cells and to investigate the possibility of internalization of the HER2/DARPin-miniSOG complex.

## EXPERIMENTAL SECTION

**Cell lines and cultivation conditions**

Human breast adenocarcinoma HER2 overexpressing SK-BR-3 cells and Chinese hamster ovary CHO cells were grown at 37°C in 5% CO_2_ in a McCoy’s 5A medium (Life technologies, USA) supplemented with 10% fetal bovine serum (HyClone, Belgium).

**Production of DARPin-miniSOG protein**

The coding sequence of the targeting module DARPin_9-29 was amplified from plasmid pCG-Hnse- DARPin-d18-9-29 (kindly provided by Dr. Pluckthun, University of Zurich). The amplified fragment was digested by NdeI and HindIII endonucleases and inserted into the pET22b expression vector digested with the same enzymes. The coding sequence of the cytotoxic module miniSOG was amplified from plasmid pSD- 4D5scFv-miniSOG [11], digested with HindIII and XhoI endonucleases and cloned into a pET22b vector in the same reading frame with a DARPin_9-29 coding sequence. The resulting expression cassette consists of inducible promoter T7 and polynucleotide sequences encoding DARPin_9-29, miniSOG, and hexahistidine tag.

The DARPin-miniSOG protein was produced in *E. coli *strain BL21(DE3). Protein expression was induced with 1 mM IPTG at OD_600_= 0.5–0.7. Cell culture was grown at 25°C for 8 h. DARPin-miniSOG was isolated from a soluble fraction by metal affinity chromatography according to the manufacturer’s instructions.

**Flow cytometry**

Flow cytometry analysis was performed on a BD Accuri C6 cytometer (Becton Dickinson, USA). Adherent cells were detached by incubation in a Versen solution (“PanEko”, Russia) and washed with PBS (“PanEko”, Russia). To determine live and dead cell subpopulations, the samples were incubated in 100 μl PBS with propidium iodide (PI, Sigma-Aldrich, USA) in final concentration 2.5 mg/ml on ice for 5 min in the dark. A minimum of 10,000 events were collected for each sample. Cells were gated on single cell populations followed by gating on viable cells (PI negative). The following detection parameters were used: 20 mV laser power, 533/30 nm band pass filter (FL1-channel) for DARPin-miniSOG, and 585/40 nm band pass filter (FL2-channel) for PI. Data were analyzed using the BD Accuri C6 software.

**Competitive binding assay**

The binding specificity of the fusion protein DARPin-miniSOG to HER2 was tested by flow cytometry. HER2 overexpressing SK-BR-3 cells were treated with DARPin-miniSOG in the presence or in the absence of a competitive agent, and the fluorescence intensity was measured. Confluent SK-BR-3 cells were detached by incubation in Versen solution, washed twice in PBS. Each sample included ~105cells. The cells were incubated with DARPin-miniSOG (500 nM) on ice for 30 min and washed twice with cold PBS to remove the unbound protein. Fluorescence intensity was detected in the FL1 channel (green fluorescence). All data were obtained as means of FL1 fluorescence intensities. DARPin_9-29 was used as a competitive agent in equimolar concentration. The anti-HER2 4D5-scFv, specific for a different HER2 epitope, was used as a negative control.

**Internalization of HER2 upon interaction with DARPin-miniSOG**

SK-BR-3 cells were grown in a McCoy’s 5A medium (Life technologies, USA) with 1% fetal bovine serum (HyClone, Belgium) for 14 h. The culture medium was removed, the cells harvested and washed twice with PBS. A cell suspension of 4 × 10^5^ cells was incubated with 250 μl of 1 μM DARPin-miniSOG in PBS on ice for 30 min, harvested by centrifugation at 4°C for 5 min at 800g, washed once with cold PBS, and aliquoted into four portions. The first portion was subjected to detection of the level of DARPin-miniSOG fluorescence on the surface of SK-BR-3 cells at 4°C. The level of cellular autofluorescence was established on untreated cells (control).

The other three portions (1 × 10^5^ cells each), treated with DARPin-miniSOG at 4°C, were incubated in a McCoy’s medium with 1% serum in a 24-well plate in 5% CO_2_ at 37°C until needed. After 4, 8, and 12 h following the first measurement, the cells were detached with a Versen solution, washed twice with PBS, and divided in half. The first half of cells was used for measuring background fluorescence; the second half was incubated in 50 μl of 1 μM DARPin-miniSOG on ice for 30 min. After incubation, the cells were once washed with cold PBS. DARPin-miniSOG fluorescence was measured for each pair of samples at each time point.

**Time course of fluorescence intensity of HER2-truncated SK-BR-3 cells treated with DARPin-miniSOG**

SK-BR-3 cells were grown in a McCoy’s 5A medium (Life technologies, USA) with 1% fetal bovine serum (HyClone, Belgium) for 14 h. The culture medium was removed, the cells harvested and washed twice with sterile PBS. To remove the extracellular domain of the HER2 receptor, a suspension of 1.5 × 10^6^ cells was incubated with 1% papain (AppliChem, Germany) at 37°C for 15min. The cells were washed with cold PBS and divided into seven portions. The first portion (the starting time point) was divided in half: the first half (~12.5 × 10^4^ cells) was stained with DARPin-miniSOG (1 μM) at 4°C, the second half was used for autofluorescence normalization. The other six portions were incubated in a McCoy’s medium with 1% serum in a 24-well plate in 5% CO_2_ at 37°C and evaluated for fluorescence at the time points of 2, 4, 8, 12, 24 and 72 h. At each time point, the cells were detached with a Versen solution, washed with cold PBS, and divided in half. The first half was treated with DARPin-miniSOG, as described above, and the second half was left untreated. Green fluorescence was measured for each pair of samples at each time point.

## RESULTS AND DISCUSSION

A targeted protein DARPin-miniSOG for selective elimination of human cancer cells under light irradiation was engineered at the laboratory of Dr. Deyev (IBCh RAS). A nonimmunoglobulin protein DARPin_ 9-29 recognizing HER2 with high affinity [6] was used as a targeting module in fusion protein DARPinminiSOG. In contrast to the anti-HER2 4D5scFv that binds subdomain IV of the HER2 extracellular domain, DARPin_9-29 binds to subdomain I of HER2 [12]. The recombinant flavoprotein miniSOG, which is known to generate reactive oxygen species under blue light irradiation [10], was used as a cytotoxic module in fusion protein DARPin-miniSOG. Being in activated state, miniSOG emits green fluorescence (λmax= 500 nm). So binding of DARPin-miniSOG to cells can be directly detected by flow cytometry.

Photoactivated toxin DARPin-miniSOG exerts a specific cytotoxic effect on HER2-positive cells, causing necrosis of irradiated cells *in vitro*.

In this study, we investigated DARPin-miniSOG interaction with HER2-overexpressing cancer cells and established if the cytotoxic module miniSOG in the fusion protein DARPin-miniSOG influenced the HER2-binding specificity of the targeted domain DARPin_9-29.

The binding specificity of targeting module DARPin_ 9-29 in the fusion protein was analyzed in a competitive inhibition assay by labeling SK-BR-3 cells expressing ~106 HER2 molecules per cell with the fluorescent fusion protein DARPin-miniSOG. Parental DARPin_9-29 was used as a competitive agent. SK-BR-3 cells were treated with DARPin-miniSOG (500 nM) or with an equimolar mixture of DARPinminiSOG (500nM) and competitive agent DARPin_9-29 (500 nM). To quantify the fluorescence level, SK-BR-3 cells were incubated with the protein at 4°C: incubation at low temperature prevents the internalization of the complex receptor-protein.

**Fig. 1:**
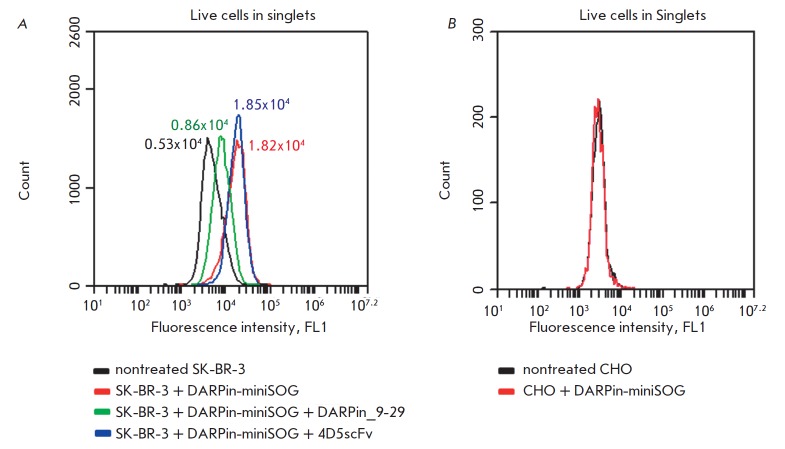
DARPin-miniSOG specific interaction with human breast adenocarcinoma HER2 overexpressing SK-BR-3 cells.** A**. Flow cytometry analysis of HER2-positive SK-BR-3 cells. Cells were treated with: DARPin-miniSOG protein (red), DARPin-miniSOG protein in combination with competitive agent DARPin_9-29 (green), and DARPin-miniSOG protein in combination with non-competitive HER2-specific mini antibody 4D5scFv (blue). The autofluorescence of untreated SKBR- 3 cells is indicated with a black line. The means of fluorescence in the green channel (Mean FL1-A) for each sample are shown in the corresponding color near the peak. **B**. Flow cytometry analysis of HER2-negative CHO cells treated with DARPin-miniSOG (red line) and not treated cells (black line)

We found that the mean fluorescence intensity of the cells treated with DARPin-miniSOG was 2-fold higher than that of the cells incubated with DARPin_9-29 and DARPin-miniSOG (*Fig. 1A*, red and green lines, respectively); i.e., DARPin_9-29 competes with DARPinminiSOG for binding to SK-BR-3 cells. Importantly, the use of 4D5scFv, which recognizes a HER2 epitope different from that of DARPin_9-29, does not result in fluorescence decline (*Fig. 1A*, blue line). HER2-negative CHO cells show no fluorescence signal following incubation with DARPin-miniSOG (*Fig. 1B*). Overall, we showed that the targeted fusion protein DARPin-miniSOG selectively binds to human breast adenocarcinoma HER2 overexpressing cells. The presence of the cytotoxic module miniSOG in the fusion protein does not affect the functional qualities of the HER2 targeting module.

To gain insight into the interactions of the targeted phototoxin DARPin-miniSOG with tumor cells, we tracked the pathway of the receptor-protein complex following binding of DARPin-miniSOG with HER2- positive cells. We were concerned as to whether the bound HER2 would be internalized. If so, what is the post endocytic traffic of HER2 after ligand binding: recycling or degradation in late lysosomes?

Internalization of HER2 has become the focus of intense research. In general, internalization mechanisms of activated receptors of the epidermal growth factor receptor family (EGFR/HER1 and HER3) have been described in detail: receptor mediated endocytosis occurs after ligand binding [13]. After internalization the complex of receptor-ligand can undergo sorting in early endosomes (fast recycling) or in multivesicular bodies (slow recycling). In both cases the receptor recycles back to the cell surface. But there exists a third pathway for the receptor-ligand complex: degradation in lysosomes. The receptor pathway is determined by the sensitivity of a receptor-ligand complex to acidic degradation during traffic from endosomes to lysosomes. Less stable complexes dissociate early, which is followed by receptors recycling to the cell membrane. More stable complexes dissociate later, and receptors in a complex with ligands degrade in lysosomes [14].

In the current literature two different points of view on HER2 internalization exist. While several studies in dicate that HER2 is endocytosis-resistant [15, 16], other studies indicate that HER2 is internalized and recycled back to the cell surface via an early endosome [17-19].

It is well established that, unlike other family members, HER2 has no natural ligands and lacks an internalization signal in its intracellular domain [20]. Therefore, it is impossible to study ligand-induced endocytosis of homodimers. There is evidence suggesting that HER2 expression inhibits formation of clathrinecoated pits [16, 21]. It was shown that upon ligandinduced heterodimerization with the other members of the EGFR family, HER2 inhibits down-regulation of the dimerization partners [22]. Trastuzumab (Herceptin), a humanized monoclonal antibody widely used in targeted therapy for HER2 positive breast cancer, is not able to promote HER2 internalization alone [23], as presumed previously. However, Trastuzumab induces HER2 internalization and intracellular degradation when combined with Pertuzumab (Perjeta), another noncompetitive anti-HER2 antibody, or L26 antibody that, similar to Pertuzumab, inhibits HER2 heterodimerization with the other members of the EGFR family [24-26].

Not only full-size antibodies, but also single-chain fragments (scFvs) have been shown to promote HER2 internalization. Ivanova and colleagues [27] reported that incubation of HER2-positive BT-474 cells with the recombinant protein 4D5scFv–dibarnase, consisting of two barnase molecules (a cytotoxic ribonuclease from* Bacillus amyloliquefaciens*) and the single-chain variable fragment of humanized anti-HER2-antibody 4D5, leads to receptor removal from the cell surface at 37°C. The internalized receptor is localized in endosomes and multivesicular bodies [27]. HER2 internalization can also occur following exposure to the fusion protein DARPinmCherry, recognizing the extracellular domain of HER2 [28].

In this work we studied HER2 internalization induced by DARPin-miniSOG binding.

**Fig. 2:**
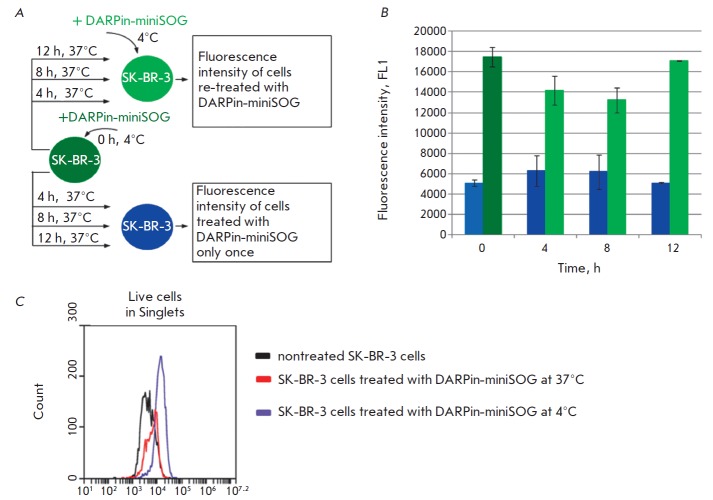
DARPin-miniSOG interaction with the HER2 on SK-BR-3 cells. **A**. Overview of the experiment. **B**. Histogram showing fluorescence of HER2-positive SK-BR-3 cells labeled with the targeted protein DARPin-miniSOG. The light blue column shows the mean autofluorescence of control SK-BR-3 cells, the deep green column shows the mean fluorescence of SK-BR-3 cells once treated with DARPin-miniSOG at 4°C. The blue columns show the means fluorescences of cells treated with DARPin-miniSOG at 4°C, followed by incubation at 37°C for 4, 8 and 12 h, respectively. The light green columns show the means fluorescences of SK-BR-3 cells treated with DARPin-miniSOG at 4°C, followed by incubation at 37°C for 4, 8 and 12 h and re-treated with DARPin-miniSOG at 4°C. The error bars indicate the standard deviations calculated from two independent experiments. **C**. Flow cytometry analysis of SK-BR-3 cells incubated with DARPinminiSOG under different conditions

The overview of the experiment is illustrated in *Fig. 2A*. The fluorescence levels of cells treated with DARPin-miniSOG at 4°C (conditions abolishing internalization) were analyzed (*Fig. 2B*, dark green column, time point 0 h) compared to autofluorescence (*Fig. 2B*, blue column, time point 0 h). Then these cells were incubated at 37°C in a CO_2_-incubator (internalization conditions). After incubation at time points 4, 8 and 12 h the fluorescence levels were measured (*Fig. 2B*, dark blue columns, time points 4, 8 and 12 h, respectively) and re-analyzed after re-treatment of cells with DARPin-miniSOG on ice (*Fig. 2B*, light green columns, time points 4, 8 and 12 h, respectively). It should be noted that the concentration of DARPinminiSOG was in excess of the receptor molecules on the cell surface.

The internalization of the complex HER2/DARPinminiSOG is suggested by a reduction in the SK-BR-3 cell fluorescence after treatment with DARPinminiSOG at 37°C as compared to the fluorescence of cells exposed to DARPin-miniSOG at 4°C: a 10-min incubation at 37°C leads to a 2-fold decrease in fluorescence intensity (*Fig. 2C*). The mean fluorescence of SK-BR-3 cells pre-treated with DARPin-miniSOG at 4°C and further incubated at 37°C (internalization conditions) is 1.4-fold higher at time point 4 h and 1.3- fold higher at time point 8 h compared to the mean background autofluorescence but returns to the initial autofluorescence at time point 12 h (*Fig. 2B*, blue columns).

The ability of SK-BR-3 cells to rebind DARPinminiSOG at 4°C compared to control SK-BR-3 cells, once treated with DARPin-miniSOG at 4°C (dark green column), declines by 1.2-fold at time point 4 h and by 1.4-fold at time point 8 h (*Fig. 2B*, light green columns). At time point 12 h the ability of SK-BR-3 cells to bind DARPin-miniSOG at 4°C completely recovers.

The ability of SK-BR-3 cells to rebind DARPinminiSOG after incubation at 37°C could result from a dissociation of the complex HER2/DARPin-miniSOG and HER2 recycling to the membrane, as well as from* de novo *synthesis of new receptor molecules.

**Fig. 3:**
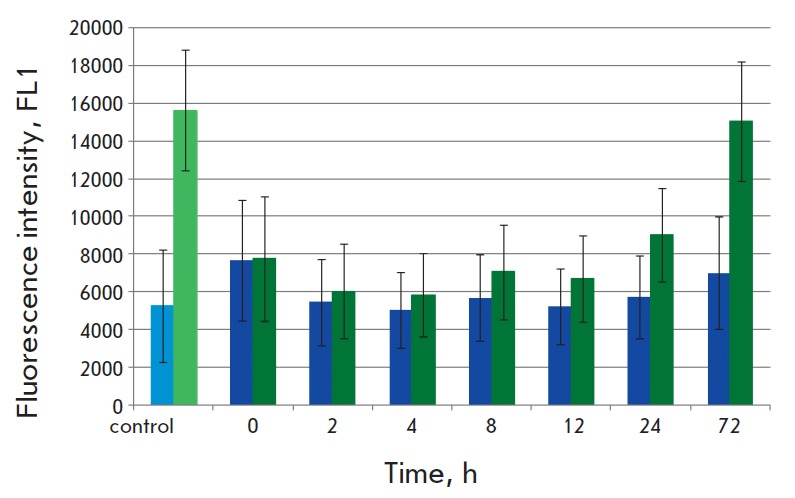
Time course of fluorescence intensity of HER2- truncated SK-BR-3 cells treated with DARPin-miniSOG. SK-BR-3 cells nontreated with papain were used as a control: the light blue column shows the mean autofluorescence of the control SK-BR-3 cells, the light green column shows the mean fluorescence of SK-BR-3 cells treated with DARPin-miniSOG at 4°C. The blue columns show the means fluorescences of SK-BR-3 cells treated with papain at each time point. The dark green columns show the means fluorescences of SK-BR-3 cells treated with papain at time point 0 h and treated with DARPin-miniSOG at 4°C at time points 2, 4, 8, 12, 24, and 72 h respectively. The error bars show the standard deviations

To estimate the contribution of *de novo *receptor biosynthesis we studied the kinetics of staining SK-BR-3 cells with DARPin-miniSOG if the extracellular domain of HER2 was truncated by papain from the cell surface. The fluorescence level of HER2-truncated SK-BR-3 cells treated with DARPin-miniSOG was 43% after 12 h and 57% after 24 h compared to the fluorescence level of HER2-positive SK-BR-3 cells treated with DARPin-miniSOG. The fluorescence level of HER2-truncated SK-BR-3 cells returned to the initial level only after 72 h (*Fig. 3*). This observation suggests that in 12 h time HER2-truncated SK-BR-3 cells fail to completely recover the HER2 receptor density on their surface (*Fig. 3*).

The results from this study show that the HER2 density on SK-BR-3 cells changes in response to stimulation: when DARPin-miniSOG interacts with HER2, the HER2/DARPin-miniSOG complex internalizes, which leads to a decrease in the number of HER2 molecules on the cell surface and, accordingly, to a decrease in the fluorescence intensity of cells re-treated with DARPinminiSOG (*Fig. 2*). After ~12 h, the mean fluorescence intensity of re-treated cells returns to its initial level. In conclusion, taking into account the experiments and dynamics of *de novo *biosynthesis of HER2 we conclude that after internalization of the HER2/DARPinminiSOG complex, its dissociation occurs and the HER2 receptor returns slowly on the cell membrane. The pool of *de novo *synthesized HER2 receptors is not significant and does not noticeably affect the mean fluorescence values of the stained cells.

## CONCLUSIONS

In this work, we have reported on the interaction of the fusion protein DARPin-miniSOG with HER2 receptors. It was found that DARPin-miniSOG induced HER2 internalization followed by recycling of HER2 back to the cell surface. These findings are important for the further development of treatment for HER2-positive cancer using a novel phototoxic protein DARPin-miniSOG.

## Abbreviations

DARPin: Designed Ankyrin Repeat Protein
HER2: human epidermal growth factor receptor 2
IPTG: isopropyl β-D-1-thiogalactopyranoside
PBS: phosphate buffered saline
PI: propidium iodide,
scFv: single-chain variable antibody fragment
